# *Acrogenospora* (Acrogenosporaceae, Minutisphaerales) Appears to Be a Very Diverse Genus

**DOI:** 10.3389/fmicb.2020.01606

**Published:** 2020-07-24

**Authors:** Dan-Feng Bao, Eric H. C. McKenzie, D. Jayarama Bhat, Kevin D. Hyde, Zong-Long Luo, Hong-Wei Shen, Hong-Yan Su

**Affiliations:** ^1^College of Agriculture and Biological Sciences, Dali University, Dali, China; ^2^Center of Excellence in Fungal Research, Mae Fah Luang University, Chiang Rai, Thailand; ^3^Department of Entomology and Plant Pathology, Faculty of Agriculture, Chiang Mai University, Chiang Mai, Thailand; ^4^Manaaki Whenua Landcare Research, Auckland, New Zealand; ^5^Retired, Goa Velha, India; ^6^Institute of Plant Health, Zhongkai University of Agriculture and Engineering, Guangzhou, China

**Keywords:** 7 new species, Acrogenosporaceae, molecular analyses, phylogeny, taxonomy

## Abstract

During a study of diversity and taxonomy of lignicolous freshwater fungi in China, nine species of *Acrogenospora* were collected. Seven of these were new species and they are described and illustrated. With morphology, additional evidence to support establishment of new species is provided by phylogeny derived from DNA sequence analyses of a combined LSU, SSU, TEF1α, and RPB2 sequence dataset. *Acrogenospora subprolata* and *A. verrucispora* were re-collected and sequenced for the first time. The genus *Acrogenospora* is far more species rich than originally thought, with nine species found in a small area of Yunnan Province, China.

## Introduction

Freshwater fungi are an ecological group that are defined by their presence in freshwater for the whole or part of their life cycle (Thomas, 1996; Wong et al., 1998), and include any species that grow on predominantly aquatic or semi-aquatic substrates (Goh and Hyde, 1996). Freshwater fungi play an important role in nutrient and carbon cycling, biological diversity and ecosystem functioning (Zhang et al., 2008; Swe et al., 2009). There have been many studies of freshwater fungi, especially on diversity, taxonomy and phylogeny (Tsui et al., 2000; Cai et al., 2002; Vijaykrishna et al., 2005; Vijaykrishna and Hyde, 2006; Hirayama et al., 2010; Ferrer et al., 2011; Barbosa et al., 2013; Raja et al., 2013, 2015) and recently from China (Hyde et al., 2016; Yang et al., 2017; Huang et al., 2018a, b; Su et al., 2018; Guo et al., 2019; Luo et al., 2019). In this study, we report nine Acrogenosporaceae species that were collected from freshwater habitats in China. *Acrogenosporaceae* was established by Jayasiri et al. (2018) to accommodate *Acrogenospora* within Minutisphaerales, with the latter being a freshwater ascomycetes order, comprising two families, Acrogenosporaceae and Minutisphaeraceae (Wijayawardene et al., 2020). Members of these two families are mostly reported from freshwater habitats (Goh et al., 1998; Raja et al., 2015; Bao et al., 2019; Hyde et al., 2019).

*Acrogenospora* was established by Ellis (1971) with two species, *Acrogenospora sphaerocephala*, and *Farlowiella carmichaeliana* (asexual morph). Ellis (1972) included two other species *A. setiformis* and *F. australis* in this genus. Hughes (1978) accepted the genus and added two additional species, *A. gigantospora* and *A. novae-zelandiae*. A taxonomic revision was provided by Goh et al. (1998) who accepted eight species, including two new combinations and two new species, and provided descriptions, illustrations and a key to species. Currently, 13 species are included in *Acrogenospora* (Hu et al., 2010; Ma et al., 2012; Hyde et al., 2019). *Acrogenospora* species are characterized by macronematous, mononematous, simple, brown, sometimes percurrently proliferating conidiophores; monoblastic, terminal or intercalary conidiogenous cells; and globose, ellipsoid or obovoid, olivaceous to brown conidia (Hughes, 1978; Goh et al., 1998).

The sexual morph of *Acrogenospora* has been linked with *Farlowiella*. Mason (1941) showed the connection between *A. megalospora* and *Farlowiella armichaeliana* based on cultural studies. Ellis (1971) reported the asexual morph of *F. carmichaeliana* as *A. carmichaeliana*. Ellis (1972) introduced *A. australis* as the asexual morph of *F. australis* based on morphological characters. Goh et al. (1998) accepted these two asexual morphs of *Farlowiella* and synonymized *A. megalospora* under *F. carmichaeliana* and *A. altissima* under *F. australis*. Jayasiri et al. (2018) carried out phylogenetic analyses with seven isolates of *Acrogenospora* and showed that *A. sphaerocephala* clustered with the sexual morph *Farlowiella carmichaeliana*. This confirmed the connection between *Acrogenospora* and *Farlowiella*. Hyde et al. (2019) also supported the asexual-sexual connection between these two genera based on a phylogenetic study. Based on recent nomenclatural changes with regards to one fungus one name, *Acrogenospora* was given priority (Wijayawardene et al., 2014; Rossman et al., 2015).

During our investigation of freshwater fungi on submerged wood along a north/south gradient in the Asian/Australasian region (Hyde et al., 2016), nine isolates of *Acrogenospora* were collected from freshwater habitats in China. Among them, two are identified as existing species, *A. subprolata* and *A. verrucispora*, and another seven are introduced as new species by comparing their morphology with known species of the genus, as well as performing phylogenetic analyses of on LSU, SSU, TEF1α, and RPB2 DNA sequence data. The objectives of this study are as follows: (i) describe and illustrate the newly collected *Acrogenospora* spp. from freshwater habitats in China; (ii) provide molecular data for *Acrogenospora* species and understand their phylogenetic relationships.

## Materials and Methods

### Isolation and Morphology

Samples of submerged wood were collected from Yunnan and Tibet provinces, China and taken to the laboratory in plastic bags. The samples were incubated in plastic boxes lined with moistened tissue paper at room temperature for one week. Specimen observations and morphological studies were conducted following the protocols provided by Luo et al. (2018).

Single spore isolations were carried out following the method described in Chomnunti et al. (2014). Germinating conidia were transferred aseptically to PDA and MEA plates supplemented with 100 mg of streptomycin and grown at room temperature in daylight. Colony color and other characters were observed and measured after 1 week and again after 3 weeks. The specimens were deposited in the Mae Fah Luang University (MFLU) Herbarium, Chiang Rai, Thailand. Living cultures are deposited in the Culture Collection at Mae Fah Luang University (MFLUCC). Facesoffungi numbers (FoF) were acquired as in Jayasiri et al. (2015) and Index Fungorum (2020). New species are established following recommendations outlined by Jeewon and Hyde (2016).

### DNA Extraction, PCR Amplification, and Sequencing

Fungal mycelium was scraped from the surface of colonies grown on potato dextrose agar (PDA) or malt extract agar (MEA) at 25°C for 4 weeks, transferred into a 1.5 mL centrifuge tube and ground using liquid nitrogen. The EZ geneTM fungal gDNA kit (GD2416) was used to extract DNA from the ground mycelium according to the manufacturer’s instructions. Primers for PCR amplification used were LSUrDNA = LR0R/LR7 (Vilgalys and Hester, 1990), SSUrDNA = NS1/NS4 (White et al., 1990), (TEF1-α) = 983F/2218R and (RPB2) = fRPB2-5F/fRPB2-7cR (Liu et al., 1999). The PCR mixture was prepared as follows: 12.5 μl of 2 × Power Taq PCR MasterMix, 20 mM Tris-HCl pH 8.3, 100 Mm KCl, 3 mM MgCl_2_, stabilizer, and enhancer), 1 μl of each primer, 1 μl genomic DNA extract and 9.5 μl deionized water. The PCR of LSU, SSU and TEF1α gene was processed as follows: 94°C for 3 min, followed by 35 cycles of denaturation at 94°C for 30 s, annealing at 56°C for 50 s, elongation at 72°C for 1 min and a final extension at 72°C for 10 min, and finally kept at 4°C. The RPB2 gene region was amplified with an initial denaturation of 95°C for 5 min, followed by 40 cycles of denaturation at 95°C for 1 min, annealing at 54°C for 40 s, elongation at 72°C for 90 s, and the final extension at 72°C for 10 min. PCR amplification was confirmed on 1% agarose electrophoresis gels stained with ethidium bromide. Purification and sequencing of PCR products were carried out using the above-mentioned PCR primers at Beijing Tsingke Biological Engineering Technology and Services Co., Ltd. (Beijing, P.R. China).

### Molecular Phylogenetic Analyses

#### Sequencing and Sequence Alignment

Sequences were assembled with BioEdit and those with high similarity indices were determined from a BLAST search to find the closest matches with taxa in *Acrogenospora* and from recently published data (Jayasiri et al., 2018; Hyde et al., 2019). All consensus sequences and the reference sequences were automatically aligned with MAFFT v. 7 and the strategy was using Auto (Katoh and Standley, 2013)^1^. Aligned sequences of each gene region (LSU, SSU, TEF1α and RPB2) were combined and manually improved using BioEdit v. 7.0.5.2 (Hall, 1999). Ambiguous regions were excluded from the analyses and gaps were treated as missing data. Phylogenetic analyses were performed using Maximum Likelihood (ML) and Bayesian tree building criteria.

#### Phylogenetic Analyses

Maximum likelihood analysis was performed at the CIPRES Science Gateway v.3.3 (Miller et al., 2010)^2^ using RAxML v. 8.2.8 as part of the “RAxML-HPC2 on XSEDE” tool (Stamatakis, 2006; Stamatakis et al., 2008). All model parameters were estimated by RAxML. The final ML search was conducted using the GTRGAMMA + I model which was estimated by using MrModeltest 2.2 (Nylander, 2004), Maximum likelihood bootstrap support was calculated from 1000 bootstrap replicates.

Bayesian analysis was performed using MrBayes v 3.1.2. (Ronquist and Huelsenbeck, 2003). The model of each genes was estimated using MrModeltest 2.2 (Nylander, 2004), GTR + I + G model was the best-fit model of LSU, SSU, TEF1α and RPB2 for Bayesian analysis. Posterior probabilities (PP) (Rannala and Yang, 1996) were performed by Markov chain Monte Carlo sampling (BMCMC) in MrBayes v.3.1.2 (Liu et al., 2012). Six simultaneous Markov chains were run for 50 million generations, and trees were sampled every 5000th generation (resulting in 10,000 trees). The first 2000 trees representing the burn-in phase of the analyses were discarded and the remaining 8000 (post burning) trees were used for calculating posterior probabilities (PP) in the majority rule consensus tree (Cai et al., 2006; Liu et al., 2012).

Maximum-parsimony analyses were performed using PAUP v.4.0b10 (Swofford, 2003). Gaps were treated as missing data with the heuristic search option with 1000 random sequence additions and tree bisection reconnection (TBR) branch-swapping. Maxtrees were unlimited, branches of zero length were collapsed and all parsimonious trees were saved. The consistency indices (CI), tree length (TL), homoplasy index (HI), rescaled consistency indices (RC), retention indices (RI) were calculated for each tree. Clade stability was assessed using a bootstrap (BT) analysis with 1000 replicates, each with 10 replicates of random stepwise addition of taxa. Other details are as provided by Jeewon et al. (2002, 2003)

Phylogenetic trees were represented by FigTree v. 1.4.4 (Rambaut, 2014) and edited in Microsoft Office PowerPoint 2016 (Microsoft Inc., United States). Newly generated sequences in this study were deposited in GenBank (Table 1) and the alignment used for the phylogenetic analyses were submitted to TreeBASE^3^ under the accession number: 26373.

**TABLE 1:** GenBank numbers and culture collection accession numbers of species included in the phylogenetic study.

Taxa	Strain	GenBank accession no.				References
		LSU	SSU	RPB2	*TEF1*α	
***Acrogenospora aquatica***	**MFLUCC 16**–**0949**	**MT340732**	–	**MT367160**	**MT367152**	This study
***Acrogenospora aquatica***	**MFLUCC 20**–**0097**	–	**MT340743**	**MT367159**	**MT367151**	This study
***Acrogenospora basalicellularispora***	**MFLUCC 16**–**0992**	**MT340729**	–	–	–	This study
*Acrogenospora carmichaeliana*	MFLU 18–1130	MH606222	–	–	–	Hyde et al., 2019
*Acrogenospora carmichaeliana*	CBS 206.36	MH867287	–	–	–	Jayasiri et al., 2018
*Acrogenospora carmichaeliana*	CBS 179.73	–	GU296148	–	–	Jayasiri et al., 2018
*Acrogenospora carmichaeliana*	CBS 164.76	GU301791	GU296129	–	GU349059	Jayasiri et al., 2018
***Acrogenospora guttulatispora***	**MFLUCC 17–1674**	**MT340730**	–	**MT367157**		This study
***Acrogenospora obovoidspora***	**MFLUCC 18–1622**	**MT340736**	**MT340747**	**MT367163**	**MT367155**	This study
***Acrogenospora olivaceospora***	**MFLUCC 20–0096**	**MT340731**	**MT340742**	**MT367158**	**MT367150**	This study
*Acrogenospora sphaerocephala*	MFLUCC 16–0179	MH606222	–	MH626448	–	Hyde et al., 2019
*Acrogenospora sphaerocephala*	JX-43	KF836062	KF836061	–	–	Jayasiri et al., 2018
*Acrogenospora sphaerocephala*	FMR11021	HF677191	–	–	–	Jayasiri et al., 2018
***Acrogenospora submersa***	**MFLUCC 18–1324**	**MT340735**	**MT340746**	**MT367162**	**MT367154**	This study
***Acrogenospora subprolata***	**MFLUCC 18–1314**	**MT340739**	**MT340750**	–	–	This study
*Acrogenospora thailandica*	MFLUCC 17–2396	MH606223	MH606221	MH626449	–	Hyde et al., 2019
***Acrogenospora verrucispora***	**MFLUCC 20–0098**	**MT340737**	**MT340748**	–	–	This study
***Acrogenospora verrucispora***	**MFLUCC 18–1617**	**MT340738**	**MT340749**	**MT367164**	**MT367156**	This study
***Acrogenospora yunnanensis***	**MFLUCC 20–0099**	**MT340734**	**MT340745**	**MT367161**	**MT367153**	This study
***Acrogenospora yunnanensis***	**MFLUCC 18–1611**	**MT340733**	**MT340744**	–	–	This study
*Acrospermum adeanum*	M 133	EU940104	EU940031	EU940320	–	Stenroos et al., 2010
*Acrospermum compressum*	M 151	EU940084	EU940012	EU940301	–	Stenroos et al., 2010
*Acrospermum graminum*	M 152	EU940085	EU940013	EU940302	–	Stenroos et al., 2010
*Aigialus grandis*	BCC 20000	GU479775	GU479739	–	GU479839	Suetrong et al., 2009
*Aigialus grandis*	BCC 18419	GU479774	GU479738	–	GU479838	Suetrong et al., 2009
*Aigialus grandis*	BCC 33563	GU479776	GU479741	–	GU479840	Suetrong et al., 2009
*Aliquandostipite khaoyaiensis*	CBS 118232	GU301796	AF201453	FJ238360	GU349048	Schoch et al., 2009
*Aliquandostipite siamensis*	SS 81.02	EF175666	EF175645	–	–	Campbell et al., 2007
*Anteaglonium abbreviatum*	GKM 1029	GQ221878	–	–	GQ221915	Mugambi and Huhndorf, 2009a
*Arthrographis arxii*	IFM 52652	AB213438	–	–	–	Giraldo et al., 2014
*Arthrographis kalrae*	CBS 693.77	AB116544	–	–	–	Giraldo et al., 2014
*Arthrographis longispora*	UTHSC 05–3220	HG004540	–	–	–	Giraldo et al., 2014
*Ascocratera manglicola*	BCC 09270	GU479782	GU479747	–	GU479846	Giraldo et al., 2014
*Asterina cestricola*	TH 591	GU586215	GU586209	–	–	Hofmann et al., 2010
*Asterina fuchsiae*	TH 590	GU586216	GU586210	–	–	Hofmann et al., 2010
*Asterina phenacis*	TH 589	GU586217	GU586211	–	–	Hofmann et al., 2010
*Asterina weinmanniae*	TH 592	GU586218	GU586212	–	–	Hofmann et al., 2010
*Asterina zanthoxyli*	TH 561	GU586219	GU586213	–	–	Hofmann et al., 2010
*Asterotexis cucurbitacearum*	VIC 24814	KP143734	–	–	–	Guatimosim et al., 2015
*Asterotexis cucurbitacearum*	PMAM 0141224	HQ610510	–	–	–	Guatimosim et al., 2015
*Astrosphaeriella fusispora*	MFLUCC 10–0555	KT955462	KT955443	–	KT955425	Phookamsak et al., 2015
*Astrosphaeriella stellata*	MAFF 239487	AB524592	AB524451	–	–	Tanaka et al., 2009
*Botryosphaeria dothidea*	CBS 115476	NG_027577	DQ677998	–	DQ767637	Phillips et al., 2008
*Capnodium salicinum*	CBS 131.34	DQ678050	DQ677997	KT216553	–	Schoch et al., 2006
*Cenococcum geophilum*	1/1/2005	JN860134	JN860120	JN860087	JN860113	Spatafora et al., 2012
*Cladosporium cladosporioides*	CBS 170.54	AY213694	DQ678004	–	–	Rakeman et al., 2005
*Dacampia hookeri*	Hafellner 73897	KT383792	–	–	–	Ertz et al., 2015
*Delitschia chaetomioides*	SMH 3253.2	GU390656	–	–	GU327753	Mugambi and Huhndorf, 2009b
*Delitschia winteri*	CBS 225.62	DQ678077	DQ678026	DQ677975	DQ677922	Schoch et al., 2006
*Diploschistes ocellatus*	AFTOL 958	AY605077	AF038877	DQ366253	–	Lumbsch et al., 2004
*Dissoconium aciculare*	CBS 204.89	GU214419	GU214523	KX288435	–	Crous et al., 2009
*Dyfrolomyces rhizophorae*	JK 5349A	GU479799	GU479766	–	GU479860	Suetrong et al., 2009
*Dyfrolomyces sinensis*	MFLUCC 17–1344	MG836699	MG836700	–	–	Hyde et al., 2018
*Dyfrolomyces tiomanensis*	NTOU3636	KC692156	KC692155	–	KC692157	Pang et al., 2013
*Eremomyces bilateralis*	CBS 781.70	HG004545	–	–	–	Giraldo et al., 2014
*Fissuroma maculans*	MFLUCC 10–0886	JN846724	JN846734	–	–	Liu et al., 2011
*Flavobathelium epiphyllum*	MPN67	GU327717	JN887382	–	JN887423	Nelsen et al., 2009
*Gloniopsis praelonga*	CBS 112415	FJ161173	FJ161134	FJ161113	FJ161090	Boehm et al., 2009
*Glonium stellatum*	CBS 207.34	FJ161179	FJ161140	–	FJ161095	Boehm et al., 2009
*Hysterium angustatum*	CBS 236.34	FJ161180	GU397359	FJ161117	FJ161096	Boehm et al., 2009
*Hysterobrevium smilacis*	CBS 114601	FJ161174	FJ161135	FJ161114	FJ161091	Boehm et al., 2009
*Jahnula aquatica*	R 68–1	EF175655	EF175633	–	–	Campbell et al., 2007
*Jahnula seychellensis*	SS2113	EF175665	EF175644	–	–	Campbell et al., 2007
*Leptoxyphium cacuminum*	MFLUCC 10–0049	JN832602	JN832587	–	–	Chomnunti et al., 2011
*Lophiotrema lignicola*	CBS 122364	GU301836	GU296166	–	GU349072	Schoch et al., 2009
*Manglicola guatemalensis*	BCC 20156	FJ743448	FJ743442	–	–	Suetrong et al., 2010
*Manglicola guatemalensis*	BCC 20079	FJ743449	FJ743443	–	–	Suetrong et al., 2010
*Massaria anomia*	CBS 591.78	GU301839	GU296169	GU371769	–	Voglmayr and Jaklitsch, 2011
*Massaria gigantispora*	M 26	–	HQ599447	–	HQ599337	Voglmayr and Jaklitsch, 2011
*Massaria inquinans*	M 19	–	HQ599444	HQ599460	HQ599342	Voglmayr and Jaklitsch, 2011
*Minutisphaera aspera*	G427–1a	KP309993	KP309999	–	–	Raja et al., 2015
*Minutisphaera fimbriatispora*	A242–8a	HM196367	HM196374	–	–	Raja et al., 2013
*Minutisphaera japonica*	JCM 18560	AB733440	AB733434	–	–	Raja et al., 2013
*Minutisphaera parafimbriatispora*	G156–4b	KP309997	KP310003	–	–	Raja et al., 2015
*Mytilinidion acicola*	EB O349	GU323209	GU323185	GU371757	–	Schoch et al., 2009
*Mytilinidion andinense*	CBS 123562	FJ161199	FJ161159	FJ161125	FJ161107	Boehm et al., 2009
*Mytilinidion mytilinellum*	CBS 303.34	FJ161184	FJ161144	FJ161119	FJ161100	Boehm et al., 2009
*Neoastrosphaeriella krabiensis*	MFLUCC 11–0025	JN846729	JN846739	–	–	Liu et al., 2011
*Oedohysterium insidens*	CBS 238.34	FJ161182	FJ161142	FJ161118	FJ161097	Boehm et al., 2009
*Paradictyoarthrinium diffractum*	MFLUCC 13–0466	KP744498	KP753960	–	–	Liu et al., 2015
*Phyllobathelium anomalum*	242	GU327722	JN887386	–	–	Nelsen et al., 2009
*Phyllosticta capitalensis*	CBS 226.77	KF206289	KF766300	KY855820	–	Guarnaccia et al., 2017
*Piedraia hortae*	CBS 480.64	GU214466	AY016349	KF902289	–	Crous et al., 2009
*Pseudoastrosphaeriella thailandensis*	MFLUCC 11–0144	KT955478	KT955458	–	KT955440	Phookamsak et al., 2015
*Pseudorobillarda eucalypti*	MFLUCC 12–0422	KF827457	KF827463	KF827496	–	Tangthirasunun et al., 2014
*Pseudorobillarda phragmitis*	CBS 398.61	EU754203	EU754104	–	–	Gruyter et al., 2009
*Pseudovirgaria grisea*	CPC 19134	JF957614	–	–	–	Braun et al., 2011
*Pseudovirgaria hyperparasitica*	CPC 10753	EU041824	–	–	–	Arzanlou et al., 2007
*Psiloglonium araucanum*	CBS 112412	FJ161172	FJ161133	FJ161112	FJ161089	Boehm et al., 2009
*Racodium rupestre*	L346	EU048583	EU048575	–	–	Muggiaa et al., 2008
*Racodium rupestre*	L424	EU048582	EU048577	–	–	Muggiaa et al., 2008
*Rhexothecium globosum*	CBS 955.73	HG004544	–	–	–	Giraldo et al., 2014
*Saccharata proteae*	CBS 115206	DQ377882	KF766311	–	–	Schoch et al., 2009
*Salsuginea ramicola*	KT 2597.1	GU479800	GU479767	GU479833	GU479861	Suetrong et al., 2009
*Scorias spongiosa*	CBS 325.33	KF901821	–	KT216542	–	Quaedvlieg et al., 2014
*Stictis radiata*	AFTOL 398	AF356663	U20610	AY641079	–	Lutzoni et al., 2001
*Strigula jamesii*	MPN548	JN887404	JN887388	–	JN887432	Nelsen et al., 2011
*Tetraplosphaeria sasicola*	MAFF 239677	AB524631	AB524490	–	–	Tanaka et al., 2009
*Thaxteriella inthanonensis*	MFLUCC11–0003	JN865199	–	–	–	Boonmee et al., 2011
*Triplosphaeria maxima*	MAFF 239682	AB524637	AB524496	–	–	Tanaka et al., 2009
*Tubeufia chiangmaiensis*	MFLUCC 11–0514	KF301538	KF301543	–	KF301557	Boonmee et al., 2014
*Tubeufia javanica*	MFLUCC 12–0545	KJ880036	KJ880035	–	KJ880037	Boonmee et al., 2014
*Ulospora bilgramii*	CBS 110020	DQ678076	DQ678025	DQ677974	DQ677921	Schoch et al., 2006

**The newly generated sequences are in bold.**

## Results

### Phylogenetic Analyses

**FIGURE 1:**
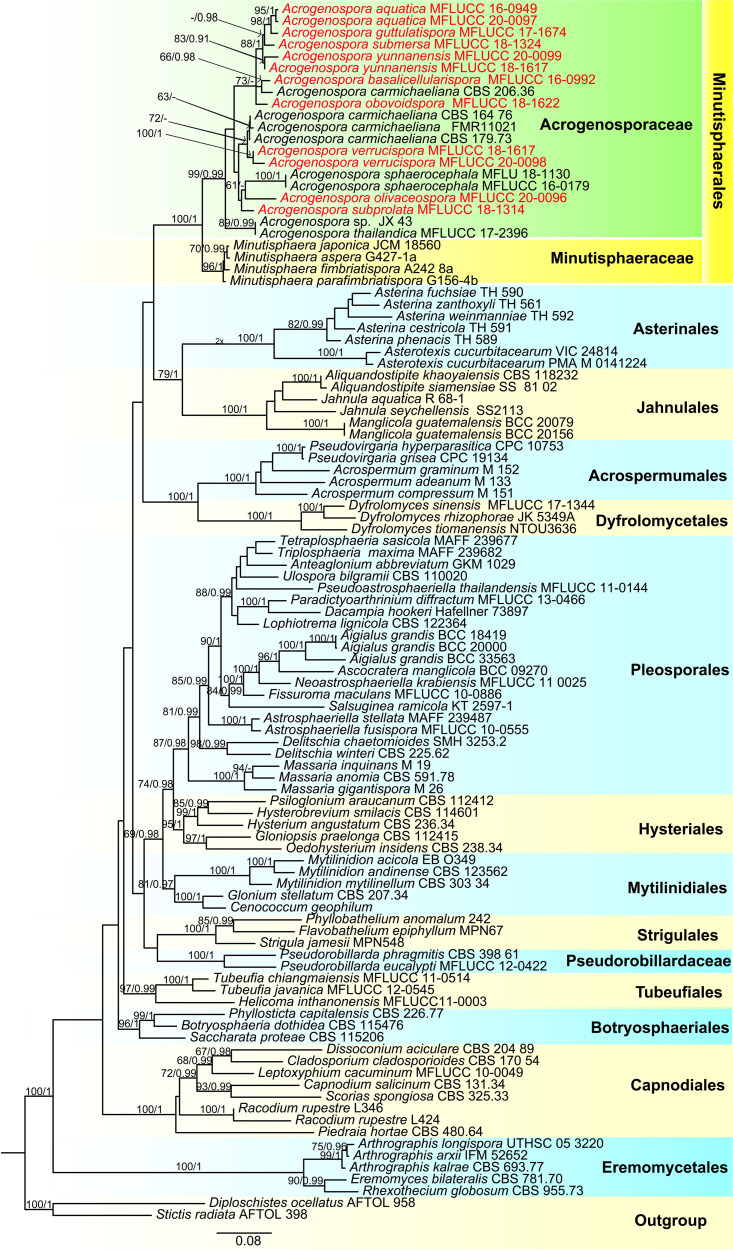
Phylogenetic tree based on RAxML analyses of a combined LSU, SSU, TEF1α and RPB2 dataset. Bootstrap support values for maximum likelihood ≥70% and Bayesian posterior probabilities ≥ 0.90 are indicated above the nodes as MLBS/PP. The tree is rooted with *Diploschistes ocellatus* (AFTOL 958) and *Stictis radiata* (AFTOL 398). The new isolates are in red.

The combined LSU, SSU, TEF, and RPB2 sequence dataset included 101 taxa (ingroup) and two outgroup taxa (*Diploschistes ocellatus* and *Stictis radiata*) with a total of 3853 characters (LSU: 867 bp; SSU:1020 bp; TEF1α: 915 bp; RPB2: 1051 bp) after alignment including the gaps. The RAxML and Bayesian analyses of the combined dataset resulted in phylogenetic reconstructions with largely similar topologies and the result of ML analysis with a final likelihood value of –49015.757408 is shown in Figure 1. The matrix had 1149 distinct alignment patterns, with 29.85% undetermined characters or gaps. Estimated base frequencies were: A = 0.253075, C = 0.235518, G = 0.278280, T = 0.194616; substitution rates AC = 1.387195, AG = 3.679854, AT = 1.133462, CG = 0.233127, CT = 7.472473, GT = 1.000000; gamma distribution shape parameter α = 0.303701. Bootstrap support values for RAxML and MP greater than 60% and Bayesian posterior probabilities greater than 0.95 are given at each node (Figure 1).

In the phylogenetic analyses all the new strains grouped with members of *Acrogenospora* within Acrogenosporaceae with high support (99% ML and 0.99 BYPP). *Acrogenospora aquatica*, *A. guttulatispora*, *A. submersa*, *A. yunnanensis* grouped together, but separated in different clades. Two isolates of *A. aquatica* (MFLUCC 16–0949 and MFLUCC 20–0097) formed a distinct clade with high statistical support (95% ML and 1 BYPP). *Acrogenospora guttulatispora* was placed as a sister taxon to *A. aquatica* and *A. submersa*. *Acrogenospora yunnanensis* clustered with *A. submersa*. *Acrogenospora basalicellularispora* clustered with *A. carmichaeliana* (CBS 206.36) and sister to *A. obovoidispora*. Two strains of *A. verrucispora* (MFLUCC 20–0098 and MFLUCC 18–1617) clustered together with high statistical support (95% ML and 1 BYPP), and sister to *A. carmichaeliana*. *Acrogenospora olivaceospora* and *A. subprolata* grouped with *A. sphaerocephala*.

***Acrogenospora aquatica*** D.F. Bao, Z.L. Luo, K.D. Hyde & H.Y. Su, **sp. nov.**

**FIGURE 2:**
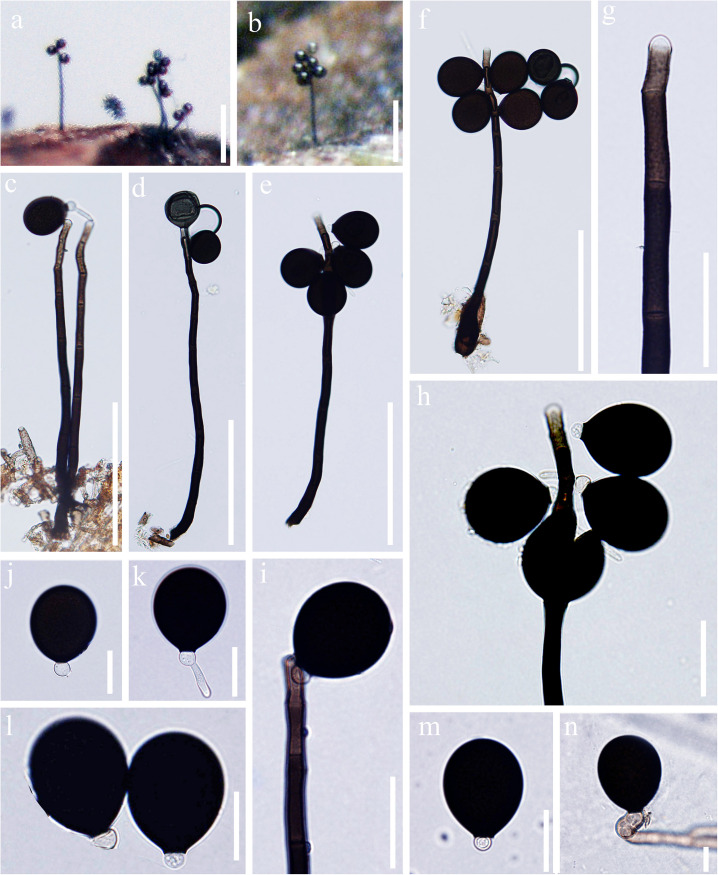
*Acrogenospora aquatica* (MFLU 20–0291, holotype) **(a,b)** Colonies on wood. **(c–f)** Conidiophores, conidiogenous cells and conidia. **(g–i)** Conidiogenous cells and conidia. **(j–m)** Conidia. **(n)** Germinating conidium. Scale bars: **(a,b)** 200 μm, **(c–f)** 100 μm, **(g–i)** 30 μm, **(j–n)** 20 μm.

*Index Fungorum number*: IF 557599; *Facesoffungi number*: FoF 07984, Figure 2

Holotype—MFLU 20–0291

Etymology—“*Aquatica*” in connection with the aquatic habitat from which it was recovered.

*Saprobic* on submerged decaying wood. **Sexual morph:** Undetermined. **Asexual morph:** *Colonies* effuse on natural substrate, hairy, dark brown. *Mycelium* mostly immersed, composed of septate, grayish brown, branched, smooth hyphae. *Conidiophores* 200–250 × 7.5–9.5 μm ($\bar{x}$ = 226 × 8.6 μm, *n* = 15), mononematous, macronematous, solitary, erect, straight or slightly flexuous, cylindrical, indeterminate, unbranched, brown to dark brown, paler toward apex, septate, smooth. *Conidiogenous cells* holoblastic, monoblastic, integrated, initially terminal, later becoming intercalary, cylindrical, smooth, pale brown, proliferating percurrently. *Conidia* 29–34.5 × 24–31 μm ($\bar{x}$ = 31.8 × 27.7 μm, *n* = 30), acrogenous, solitary, subprolate to broadly ellipsoidal, base truncate, dark brown to black, aseptate, lacking guttules, with a hyaline, globose to subglobose basal cell, smooth.

*Material examined*: CHINA, Yunnan Province, Dali, Cangshan Mountain, on decaying wood submerged in a stream, January 2016, Q.S. Zhou, S-763 (MFLU 20–0291, **holotype**), ex-type culture MFLUCC 20–0097. CHINA, Yunnan Province, Dali, Cangshan Mountain, on decaying wood submerged in Qingbixi Stream, March 2016, Z. L. Luo, S-282 (DLU 282, **isotype**), living culture MFLUCC 16-0949.

*Notes*: In our study, we found two species, *A. basalicellularispora* and *A. aquatica* with a hyaline, globose to subglobose basal cell. *Acrogenospora aquatica* can be distinguished from *A. basalicellularispora* by the size of conidiophores (259–395 × 8–12 vs. 202–250 × 7.8–9.3 μm). In addition, conidia of *A. basalicellularispora* are pale orange-brown to olivaceous-brown, with several small to large guttules, while conidia of *A. aquatica* are dark brown to black and lack guttules.

*Acrogenospora aquatica* is phylogenetically close to *A. guttulatispora. Acrogenospora aquatica* similar to *A. guttulatispora* in having mononematous, macronematous, unbranched conidiophores, holoblastic, monoblastic conidiogenous cells and acrogenous, dark brown to black conidia. However, *A. aquatica* differs from *A. guttulatispora* in having subprolate to broadly ellipsoidal conidia with a hyaline, globose to subglobose basal cell, lacking guttules, while conidia of *A. guttulatispora* are spherical or subspherical, with a large guttule, lacking basal cell.

***Acrogenospora basalicellularispora*** D.F. Bao, Z.L. Luo, K.D. Hyde & H.Y. Su, **sp. nov.**

**FIGURE 3:**
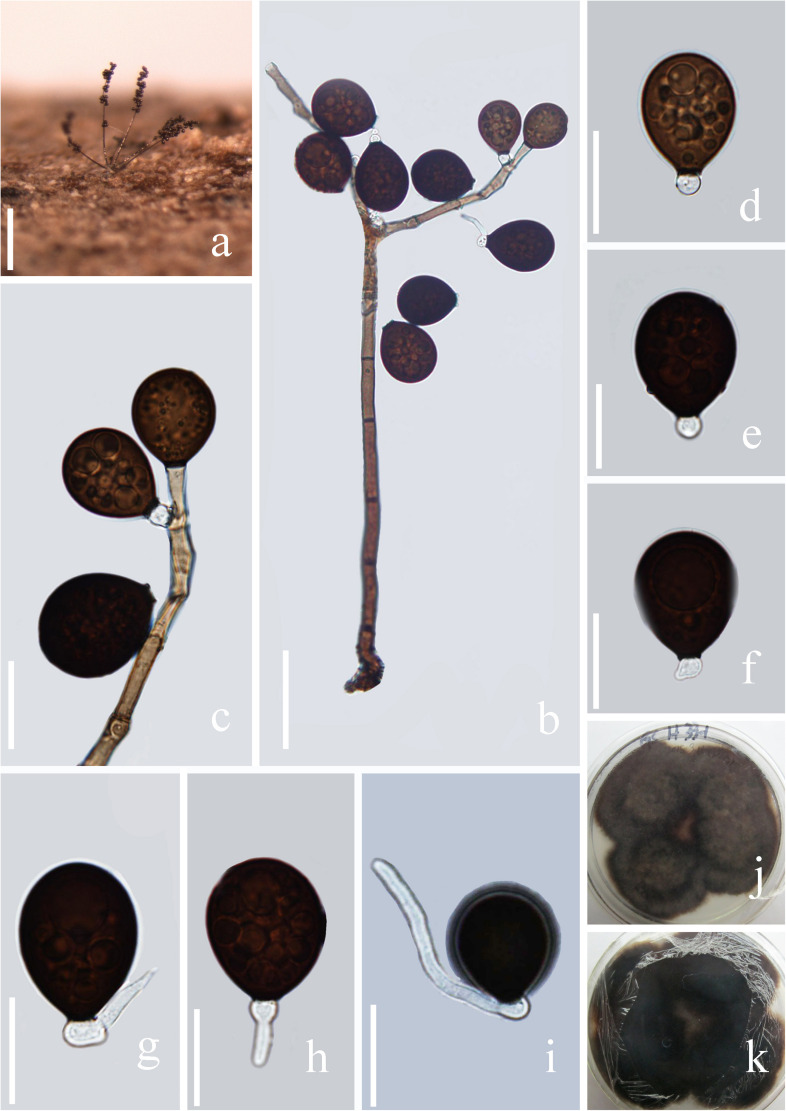
*Acrogenospora basalicellularispora* (MFLU 20–0288, holotype) **(a)** Colony on wood. **(b)** Conidiophores, conidiogenous cells and conidia. **(c)** Conidiogenous cells and conidia. **(d–h)** conidia. **(i)** Germinating conidium. **(j,k)** Culture on MEA (upper and lower view). Scale bars: **(a)** 200 μm, **(b)** 50 μm, **(c–i)** 20 μm.

*Index Fungorum number*: IF 557596; *Facesoffungi number*: FoF 07981, Figure 3

Holotype—MFLU 20–0288

Etymology—Referring to the conidia which have a basal cell.

*Saprobic* on submerged decaying wood. **Sexual morph:** Undetermined. **Asexual morph:** *Colonies* effuse on natural substrate, hairy, dark brown. *Mycelium* mostly immersed, composed of grayish brown, septate, branched, smooth hyphae. *Conidiophores* 260–395 × 8–12 μm ($\bar{x}$ = 327 × 10 μm, *n* = 20) wide, mononematous, macronematous, solitary, cylindrical, erect, straight or slightly flexuous, mostly unbranched, septate, brown to dark brown, slightly paler toward apex, smooth. *Conidiogenous cells* holoblastic, monoblastic, integrated, initially terminal, later becoming intercalary, cylindrical, smooth, pale brown, proliferating percurrently. *Conidia* 27.5–33.7 × 21.7–25.8 μm ($\bar{x}$ = 30.6 × 23.7 μm, *n* = 30) wide, acropleurogenous, solitary, dry, broadly obovoid to spherical, smooth, pale orange-brown to olivaceous brown, aseptate, with several small or large guttules, with a small, hyaline, subcylindrical to subglobose basal cell, germinating from basal cell.

*Material examined*: CHINA, Yunnan Province, Gaoligongshan Mountain, on decaying wood submerged in a stream, August 2015, A.L. Shi, S-431 (MFLU 20–0288, **holotype**); ex-type culture, MFLUCC 16–0992.

*Notes*: In the phylogenetic analysis, *Acrogenospora basalicellularispora* clustered with *A. sphaerocephala* (CBS 206.36) with low support (66% ML and 0.98 BYPP). Unfortunately, CBS 206.36 lacks a morphological description and only LSU sequence data is available in GenBank. Morphologically, our new isolate can be distinguished from other *Acrogenospora* species by its pale orange-brown to olivaceous brown, broadly obovoid to spherical conidia with several small to large guttules and a small, hyaline, subcylindrical to subglobose basal cell. In our study, *A. aquatica* also has conidia with a basal cell. However, we can distinguish them by the shape (broadly obovoid to spherical vs. subprolate to broadly ellipsoidal) and color (pale orange-brown to olivaceous brown vs dark brown to black) of conidia and size (260–395 × 8–12 vs. 200–250 × 7.5–9.5 μm) of conidiophores.

***Acrogenospora guttulatispora*** D.F. Bao, Z.L. Luo, K.D. Hyde & H.Y. Su, **sp. nov.**

**FIGURE 4:**
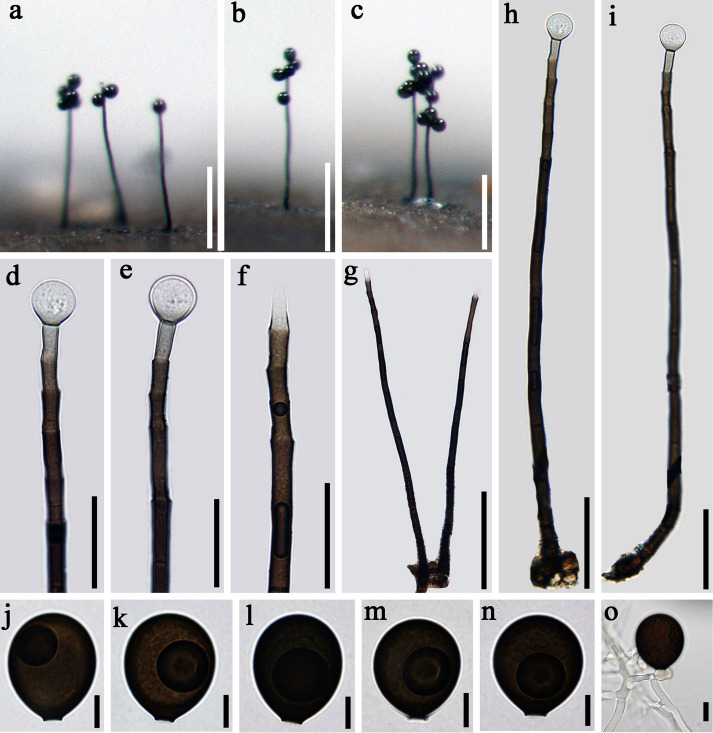
*Acrogenospora guttulatispora* (MFLU 20–0289, holotype) **(a–c)** Colonies on wood. **(d–f)** Conidiogenous cells and conidia. **(g–i)** Conidiophores. **(j–n)** conidia. **(o)** Germinating conidium. Scale bars: **(a–c)** 200 μm, **(d–f)** 30 μm, **(g)** 100 μm, **(h,i)** 50 μm, **(j–o)** 10 μm.

*Index Fungorum number*: IF 557597; *Facesoffungi number*: FoF 07982, Figure 4

Holotype—MFLU 20–0289

Etymology—Referring to the large guttule in the conidia.

*Saprobic* on submerged decaying wood. **Sexual morph:** Undetermined. **Asexual morph:** *Colonies* effuse on natural substrate, hairy, dark brown. *Mycelium* mostly immersed, composed of septate, grayish brown, branched, smooth hyphae. *Conidiophores* 295–330 × 7.5–8.5 μm ($\bar{x}$ = 312.7 × 8 μm, *n* = 15), mononematous, macronematous, solitary, erect, straight or slightly flexuous, cylindrical, indeterminate, unbranched, dark brown, paler toward apex, pale brown to hyaline at apex, septate, guttulate, smooth. *Conidiogenous cells* holoblastic, monoblastic, integrated, initially terminal, later becoming intercalary, cylindrical, smooth, pale brown, proliferating percurrently. *Conidia* 30–33.5 × 26.5–28 μm ($\bar{x}$ = 34 × 27 μm, *n* = 30), acropleurogenous, solitary, spherical or subspherical, truncate at base, hyaline when young, dark brown when mature, aseptate, with a large guttule, smooth.

*Material examined*: CHINA, Yunnan Province, Dali, Cangshan Mountain, on decaying wood submerged in Heilongxi stream, June 2013, Z.L. Luo. S-189 (MFLU 20–0289, **holotype**), ex-type culture, MFLUCC 17–1674 = ICMP 21772.

*Notes*: *Acrogenospora guttulatispora* can be distinguished from other species by the large guttule in the conidia. In the phylogenetic analyses, *A. guttulatispora* is close to *A. aquatica*. However, the conidia of *A. guttulatispora* are spherical or subspherical with a large guttule, without a basal cell. While, those *A. aquatica* are subprolate to broadly ellipsoidal with a hyaline, globose to subglobose basal cell. In addition, there are 22 base pair differences in the RPB2 region between these two species.

***Acrogenospora obovoidspora*** D.F. Bao, Z.L. Luo, K.D. Hyde & H.Y. Su, **sp. nov.**

**FIGURE 5:**
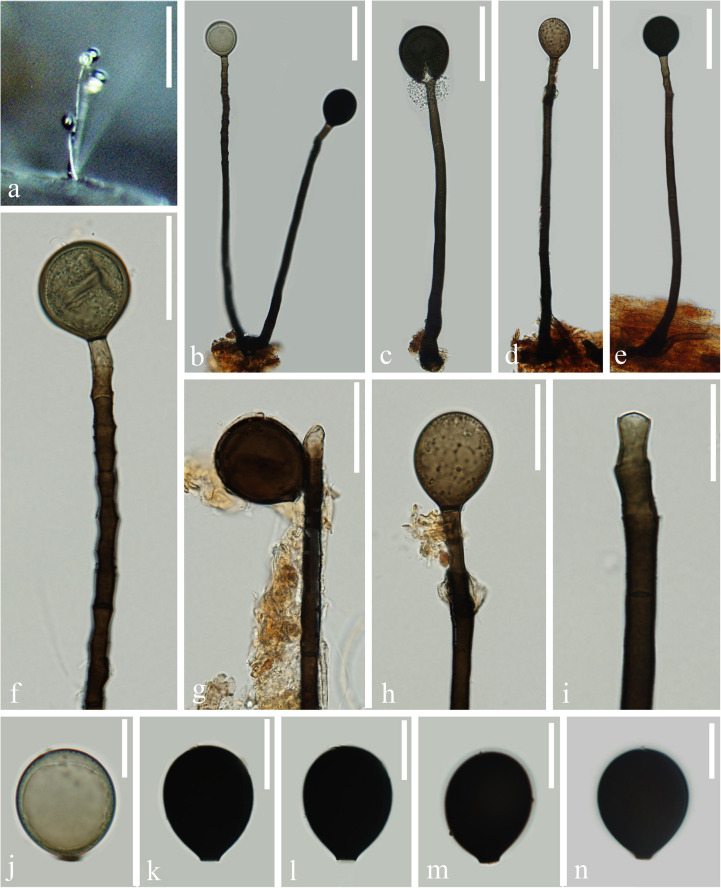
*Acrogenospora obovoidispora* (MFLU 20–0295, holotype) **(a)** Colony on wood. **(b–e)** Conidiophores with conidia. **(f–i)** Conidiogenous cells with conidia. **(j–n)** Conidia. Scale bars: **(a)** 200 μm, **(b–e)** 50 μm, **(f–h)** 30 μm, **(i–n)** 20 μm.

*Index Fungorum number*: IF 557602; *Facesoffungi number*: FoF 04691, Figure 5

Holotype—MFLU 20–0295

Etymology—Referring to the broadly obovoid conidia of this fungus.

*Saprobic* on submerged decaying wood. **Sexual morph:** Undetermined. **Asexual morph:** *Colonies* effuse on natural substrate, hairy, dark brown. *Mycelium* partly immersed, partly superficial, composed of septate, brown to dark brown, branched, smooth hyphae. *Conidiophores* 209–277 × 7.5–10 μm ($\bar{x}$ = 243 × 8.8 μm, *n* = 15), mononematous, macronematous, solitary, erect, straight or slightly flexuous, cylindrical, unbranched, brown to dark brown, paler toward apex, septate, smooth. *Conidiogenous cells* holoblastic, monoblastic, integrated, initially terminal, later becoming intercalary, cylindrical, smooth, pale brown, proliferating percurrently. *Conidia* 32.5–37.5 × 27–32 μm ($\bar{x}$ = 35 × 29.6 μm, *n* = 30) wide, acrogenous, solitary, oval to broadly ellipsoidal, base truncate, aseptate, olivaceous brown to black, thick-walled, smooth.

*Material examined*: CHINA, Yunnan Province, Dali, Huadianba Mountain, saprobic on decaying wood submerged in a stream, 9 December 2017, Z.L. Luo, S-1614 (MFLU 20–0295, **holotype**); ex-type culture, MFLUCC 18–1622.

*Notes*: *Acrogenospora obovoidispora* is similar to *A. gigantospora* in having mononematous, macronematous, conidiophores and solitary, aseptate conidia. However, *Acrogenospora obovoidspora* differs from *A. gigantospora* in having solitary conidiophores and oval to broadly ellipsoidal, olivaceous brown to black conidia, while conidiophores of *A. gigantospora* are single or in groups of 2–4, and conidia are broadly obovoid to subspherical, dark brown to black (Ma et al., 2012).

***Acrogenospora olivaceospora*** D.F. Bao, Z.L. Luo, K.D. Hyde & H.Y. Su, **sp. nov.**

**FIGURE 6:**
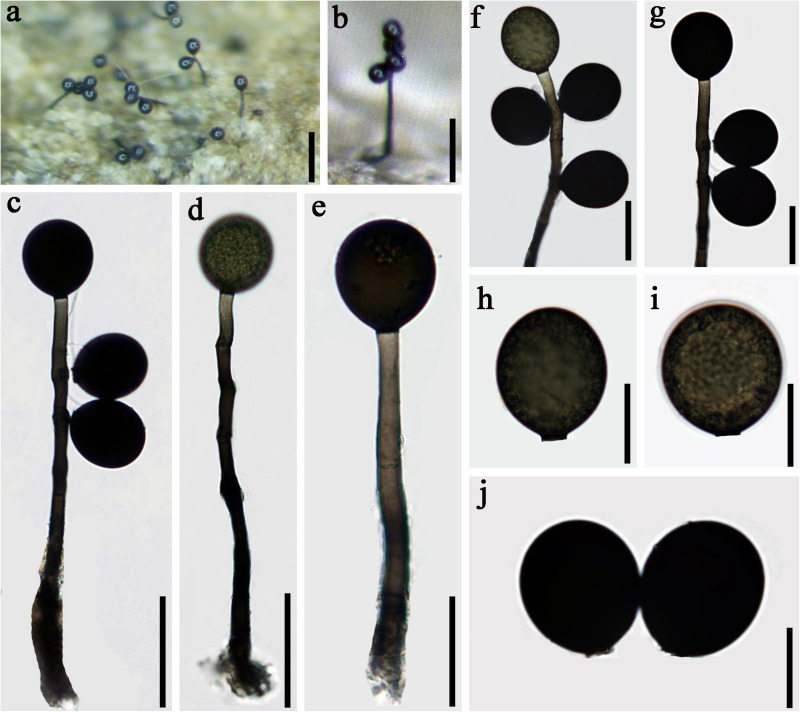
*Acrogenospora olivaceospora* (MFLU 20–0290, holotype) **(a,b)** Colonies on wood. **(d,e)** Conidiophores, conidiogenous cells, and conidia. **(f,g)** Conidiogenous cells with conidia. **(a)** 200 μm, **(b)** 100 μm, **(h–j)** Conidia. Scale bars: **(c,d)** 50 μm, **(e–g)** 30 μm, **(h–j)** 20 μm.

*Index Fungorum number*: IF 557598; *Facesoffungi number*: FoF 07983, Figure 6

Holotype—MFLU 20–0290

Etymology—Referring to the conidia which are olive-green.

*Saprobic* on submerged decaying wood. **Sexual morph:** Undetermined. **Asexual morph:** *Colonies* effuse on natural substrate, hairy, dark brown. *Mycelium* mostly immersed, composed of grayish brown, septate, branched, smooth hyphae. *Conidiophores* 100–175 × 6–9 μm ($\bar{x}$ = 137 × 7.4 μm, *n* = 20), mononematous, macronematous, solitary, erect, straight or slightly flexuous, cylindrical, indeterminate, unbranched, dark brown to olive, paler toward apex, septate, smooth. *Conidiogenous cells* holoblastic, monoblastic, integrated, initially terminal, later becoming intercalary, cylindrical, smooth, pale brown, proliferating percurrently. *Conidia* 32–37 × 28–33 μm ($\bar{x}$ = 34.5 × 30.5 μm *n* = 30), acropleurogenous, solitary, subprolate to broadly ellipsoidal, base truncate, olive to black, aseptate, thick-walled, lacking guttules, smooth.

*Material examined*: CHINA, Yunnan Province, Dali, Cangshan Mountain, on decaying wood submerged in a stream, March 2016, H.W. Shen, S-715 (MFLU 20–0290, **holotype**), ex-type culture, MFLUCC 20–0096.

*Notes*: In the phylogenetic analyses, *Acrogenospora olivaceospora* clustered with *A. sphaerocephala* (MFLU 18-1130 and MFLUCC 16-0179). However, *A. olivaceospora* differs from *A. sphaerocephala* by the shape, color and size of conidia (Table 2). *Acrogenospora olivaceospora* has olive to black, subprolate to broadly ellipsoidal conidia, lacking guttules, while conidia of *A. sphaerocephala* are olive-green to brown, spherical or subspherical and guttulate.

**TABLE 2:** Morphological comparison of *Acrogenospora* species.

Taxa	Conidiophores		Conidia			Sequence data	References
	Color	Size (μm)	Color	Shape	Size (μm)		
*Acrogenospora altissima*	Blackish brown to black	Up to 800 × 12–20	Dark to blackish brown	Broadly ellipsoidal	40–60 × 30–36	Absent	Goh et al., 1998
*A. aquatica*	Brown to dark brown, paler toward apex	202–250 × 7.5–9.5	Dark brown to black	Subprolate to broadly Ellipsoidal, with a basal cell and guttules	29–34.5 × 24.5–31	Present	This study
*A. basalicellularispora*	Brown to dark brown, paler toward apex	259–395 × 8–12	Pale orange-brown to olivaceous brown	Broadly obovoid to spherical, with basal cell	27.5–33.5 × 21.5–25.5	Present	This study
*A. carmichaeliana*	Brown to dark brown	Up to 400 × 9–12	Brown to dark brown	Broadly ellipsoidal to obovoid	19–32 × 16–23.5	Present	Goh et al., 1998
*A. ellipsoidea*	Pale orange brown to mid brown	87.5–162.5 × 6.5–7.5	Dark brown	Ellipsoidal, atrobrunnea,	32–41 × 17–24	Absent	Hu et al., 2010
*A. gigantospora*	Dark blackish brown	Up to 700 × 9–14.5	Dark brown to black (opaque)	Broadly obovoid to spherical	25–55 × 21–50	Absent	Hughes, 1978
*A. guttulatispora*	Dark brown, paler toward apex	294–331 × 7.5–8.6	Hyaline when young, dark brown at mature,	Spherical or subspherical, with a large guttule	30–33.5 × 26.5–28	Present	This study
*A. hainanensis*	Brown to dark brown, paler toward the apex	60–80 × 2–3.5	Brown	Spherical or subspherical	7.5–9.5 × 7–8.5	Absent	Ma et al., 2012
*A. megalospora*	Black (opaque), Yellow brown at apex	Up to 400 × 9–12	Mid to dark brown	Obovoid	19–32 × 13–23	Absent	Goh et al., 1998
*A. novae-zelandiae*	Black (opaque), paler at apex	Up to 720 × 10–16	Mid to dark brown	Broadly ellipsoidal to oblong	26–54 × 21.5–30.5	Absent	Hughes, 1978
*A. obovoidspora*	Brown to dark brown, paler toward apex	209–277 × 7.5–10	Olivaceous brown to black	Oval to broadly obovoid	32.4–37.6 × 27–32	Present	This study
*A. olivaceospora*	Dark brown to olive, paler toward apex	102–172 × 5.8–9	Olive to black	Subprolate to broadly ellipsoidal	32–36.9 × 28–32.8	Present	This study
*A. ovalia*	Pale to mid brown	Up to 240 × 4–4.5	Mid orange-brown	Oval to oblong or broadly obovoid	24–33 × 18–22	Absent	Goh et al., 1998
*A. setiformis*	Dark blackish brown	Up to 350 × 4–7	Dark reddish brown	Broadly ellipsoidal	14.5–24 × 10.5–19	Absent	Ellis, 1972
*A. sphaerocephala*	Mid to dark brown, pale brown at apex	100–730 × 7.2–10.5	Pale to mid brown	Subspherical	17–30 × 15.5–30	Present	Hughes, 1978
*A. submersa*	Brown to dark brown, paler toward apex	163–223 × 6.7–10	Hyaline when young, pale orange-brown to olivaceous brown at mature	Spherical or subspherical	28–32.5 × 25–28	Present	This study
*A. subprolata*	Pale to mid brown	150–300 × 9–12	Pale orange-brown to olivaceous brown	Broadly ellipsoidal to subprolate	39–46 × 30–39	Present	Goh et al., 1998
*A. thailandica*	Pale to dark brown, paler toward the apex	850–950 × 3.5–8	Olive-green to dark brown	Spherical or subspherical	15.5–24.5	Present	Hyde et al., 2019
*A. verrucispora*	Brown to dark brown, paler toward apex	100–230 × 5–6	Mid to dark brown	Spherical or subspherical	19–21.5 diam	Present	This study
*A. yunnanensis*	Brown to dark brown, paler toward apex	260–391 × 8.6–12	Hyaline when young, dark brown to black at mature	Spherical or subspherical	23–32.5 × 22–30	Present	This study

*Acrogenospora olivaceospora* is most similar to *A. subprolata* in having subprolate to broadly ellipsoidal, olive to black, aseptate, thick-walled conidia. However, *A. olivaceospora* has solitary conidiophores whereas those of *A. subprolata* are sometimes in small groups. In addition, the conidiophores of *A. olivaceospora* are shorter (102–172 × 5.8–9 vs. 150–300 × 9–12 μm) and the conidia are smaller (32–36.9 × 28–32.8 vs. 39–46 × 30–39 μm).

***Acrogenospora submersa*** D.F. Bao, Z.L. Luo, K.D. Hyde & H.Y. Su, **sp. nov.**

**FIGURE 7:**
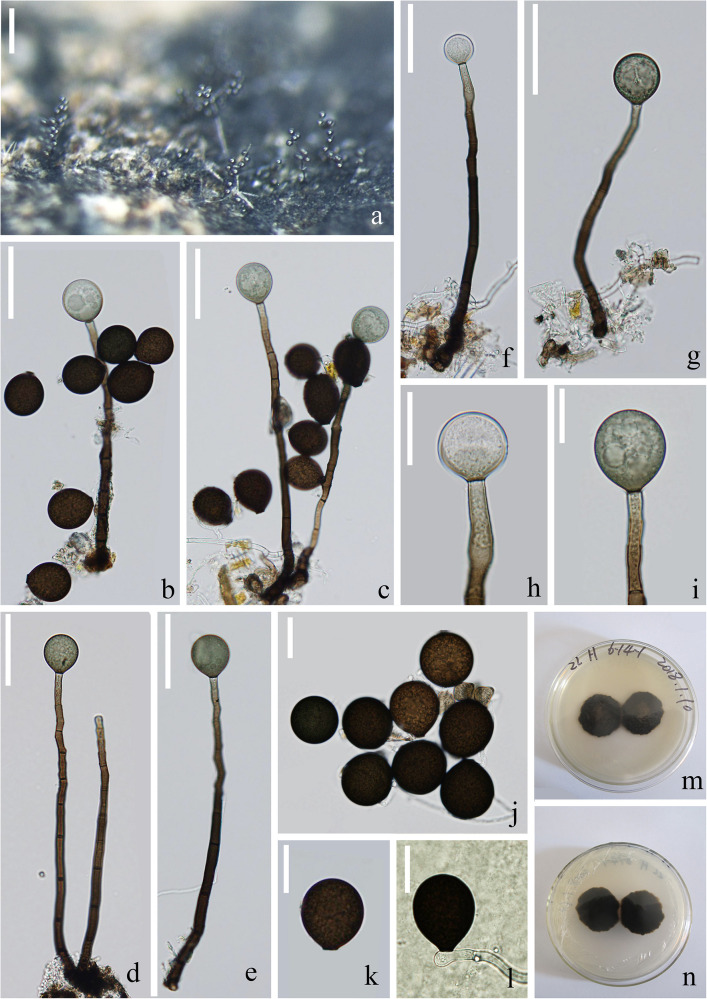
*Acrogenospora submersa* (MFLU 20–0294, holotype) **(a)** Colonies on wood. **(b–g)** Conidiophores with conidia. **(h,i)** Conidiogenous cells with conidia. **(j,k)** conidia. **(i)** Germinating conidium. **(m,n)** Culture on MEA (upper and lower view). Scale bars: **(a)** 200 μm, **(b–g)** 50 μm, **(h–l)** 20 μm.

*Index Fungorum number*: IF 557601; *Facesoffungi number*: FoF 07986, Figure 7

Holotype—MFLU 20–0294,

Etymology—Referring to the submerged habitat of this fungus.

*Saprobic* on submerged decaying wood. **Sexual morph:** Undetermined. **Asexual morph:** *Colonies* effuse on natural substrate, hairy, dark brown. *Mycelium* partly immersed, partly superficial, composed of septate, brown to dark brown, branched, smooth hyphae. *Conidiophores* 163–223 × 6.5–10 μm ($\bar{x}$ = 193 × 8.4 μm, *n* = 15), mononematous, macronematous, solitary, erect, straight or slightly flexuous, cylindrical, unbranched, brown to dark brown, paler toward apex, septate, smooth. *Conidiogenous cells* holoblastic, monoblastic, integrated, initially terminal, later becoming intercalary, cylindrical, smooth, pale brown, proliferating percurrently. *Conidia* 28–32.5 × 25–28 μm ($\bar{x}$ = 30.3 × 26.5 μm, *n* = 30), acropleurogenous, solitary, spherical or subspherical, base truncate, aseptate, hyaline when young, pale orange-brown to olivaceous brown when mature, smooth.

*Material examined*: CHINA, Yunnan Province, saprobic on decaying wood submerged in Lancang River, 9 December 2017, Z.L. Luo, S-1601 (MFLU 20–0294, **holotype**), ex-type culture, MFLUCC 18–1324.

*Notes*: *Acrogenospora submersa* is similar to *A. hainanensis* in having mononematous, macronematous, solitary, proliferating percurrently conidiophores, monoblastic, integrated, terminal conidiogenous cells and spherical or subspherical, aseptate conidia. However, *A. submersa* differs from *A. hainanensis* by having longer conidiophores (163–223 × 6.7–10 μm vs. 60–80 × 2–3.5 μm), and much larger conidia (28–32.5 × 25–28 μm vs. 7.5–9.5 × 7–8.5 μm), which are hyaline to pale orange-brown or olivaceous brown rather than brown. Phylogenetically, *A. submersa* is related to *A. guttulatispora* but in a distinct lineage. Therefore, we introduce it as a new species.

***Acrogenospora subprolata*** Goh, K.D. Hyde & C.K.M. Tsui, Mycol. Res. 102(11): 1314 (1998) Figure 8

**FIGURE 8:**
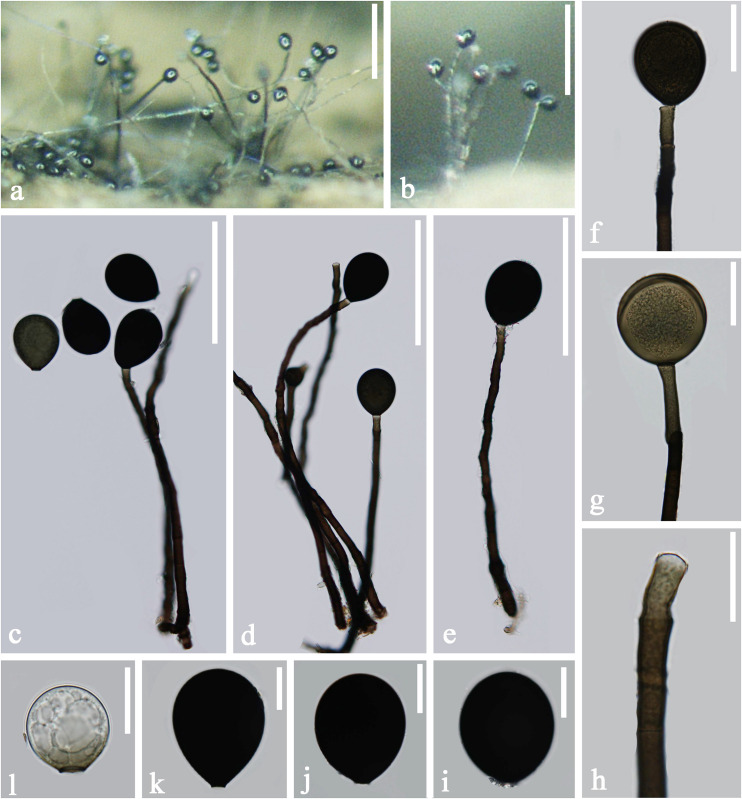
*Acrogenospora subprolata* (MFLU 20–0293) **(a,b)** Colonies on wood. **(c–e)** Conidiophores and conidia. **(f–h)** Conidiogenous cells with conidia. **(i–l)** conidia. Scale bars: **(a,b)** 200 μm, **(c–e)** 100 μm, **(f,g)** 30 μm, **(h–l)** 20 μm.

*Saprobic* on submerged decaying wood. **Sexual morph:** Undetermined. **Asexual morph:** *Colonies* effuse on natural substrate, hairy, dark brown. *Mycelium* partly immersed, partly superficial, composed of septate, brown to dark brown, branched, smooth hyphae. *Conidiophores* 212.5–348 × 8–10.5 μm ($\bar{x}$ = 280.5 × 9.4 μm, *n* = 15) wide, mononematous, macronematous, solitary, or in a small group, erect, straight or slightly flexuous, cylindrical, unbranched, brown to dark brown, paler toward apex, septate, smooth. *Conidiogenous cells* holoblastic, monoblastic, integrated, initially terminal, later becoming intercalary, cylindrical, smooth, pale brown, proliferating percurrently. *Conidia* 40–51 × 32–40 μm ($\bar{x}$ = 45 × 31.5μm, *n* = 30) wide, acrogenous, solitary, subprolate to broadly ellipsoidal, base truncate, aseptate, hyaline when young, olivaceous brown to black when mature, thick-walled, smooth.

*Material examined*: CHINA, Tibet Province, saprobic on decaying wood submerged in a stream, 2 May 2017, Z.L. Luo. S-1455 (MFLU 20–0293), living culture, MFLUCC 18–1314.

*Notes*: *Acrogenospora subprolata* is characterized by conidiophores that are macronematous, mononematous, solitary or in groups of 2–4 with multiple percurrent proliferations and by acrogenous, subprolate to broadly ellipsoidal, pale orange-brown to olivaceous brown, aseptate, thick-walled conidia. Our isolate fits well with the characters of *A. subprolata* as described by Goh et al. (1998). Therefore, we identify this collection as *A. subprolata*.

***Acrogenospora verrucispora*** Hong Zhu, L. Cai & K.Q. Zhang [as ‘verrucospora’], Mycotaxon 92: 384 (2005) Figure 9

**FIGURE 9:**
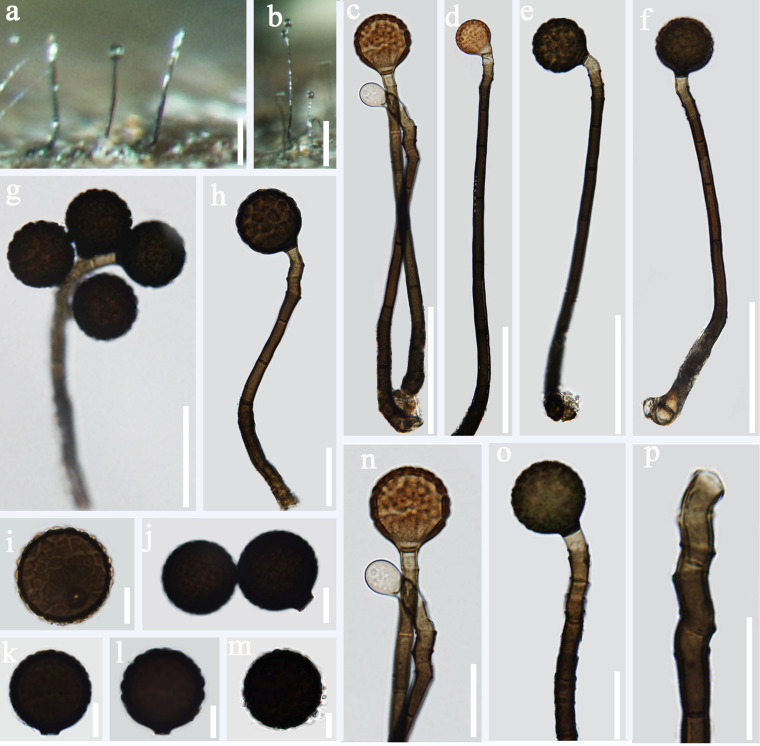
*Acrogenospora verrucispora* (MFLU 20–0287) **(a,b)** Colonies on wood. **(c–h)** Conidiophores with conidia. **(n–p)** Conidiogenous cells with conidia. **(i–m)** conidia. Scale bars: **(a–f)** 50 μm, **(g,h,n–p)** 20 μm, **(i–m)** 10 μm.

*Saprobic* on submerged decaying wood. **Sexual morph:** Undetermined. **Asexual morph:** *Colonies* effuse on natural substrate, hairy, dark brown. *Mycelium* partly immersed, partly superficial, composed of septate, brown to dark brown, branched, smooth hyphae. *Conidiophores* 103–149 × 5.6–7.4 μm ($\bar{x}$ = 125.8 × 6.5 μm, *n* = 15) wide, mononematous, macronematous, solitary or sometimes in a small group, erect, straight or slightly flexuous, cylindrical, unbranched, brown to dark brown, paler toward apex, septate, smooth. *Conidiogenous cells* holoblastic, monoblastic, integrated, initially terminal, later becoming intercalary, cylindrical, smooth, pale brown, proliferating percurrently. *Conidia* 21.3–26.5 × 20.6–25.5 μm ($\bar{x}$ = 24 × 23 μm, *n* = 30) wide, acrogenous, solitary, spherical or subspherical, base truncate, aseptate, hyaline when young, orange-brown to olivaceous brown when mature, distinctly verrucose.

*Material examined*: CHINA, Yunnan Province, Gaoligongshan Mountain, saprobic on decaying wood submerged in a stream, May 2017, H.W. Shen. S-1402 (MFLU 20–0287), living culture, MFLUCC 20–0098. S-1328 (DLU 1328), living culture, MFLUCC 18–1617.

*Notes*: *Acrogenospora verrucispora* was introduced by Zhu et al. (2005) with distinct verrucose conidia, it was collected from bamboo in a stream in Yunnan province, China. *A. verrucispora* is characterized by mononematous, macronematous, proliferating percurrently conidiophores, monoblastic, integrated conidiogenous cells and acrogenous, solitary, spherical or subspherical, verrucose conidia. Our isolate fits well with the original description of *A. verrucispora*. As the sequence data of *A. verrucispora* is not available in GenBank, we identify our isolate as *A. verrucispora* based on the morphological characters and provide sequence data for this species.

***Acrogenospora yunnanensis*** D.F. Bao, Z.L. Luo, K.D. Hyde & H.Y. Su, **sp. nov.**

**FIGURE 10:**
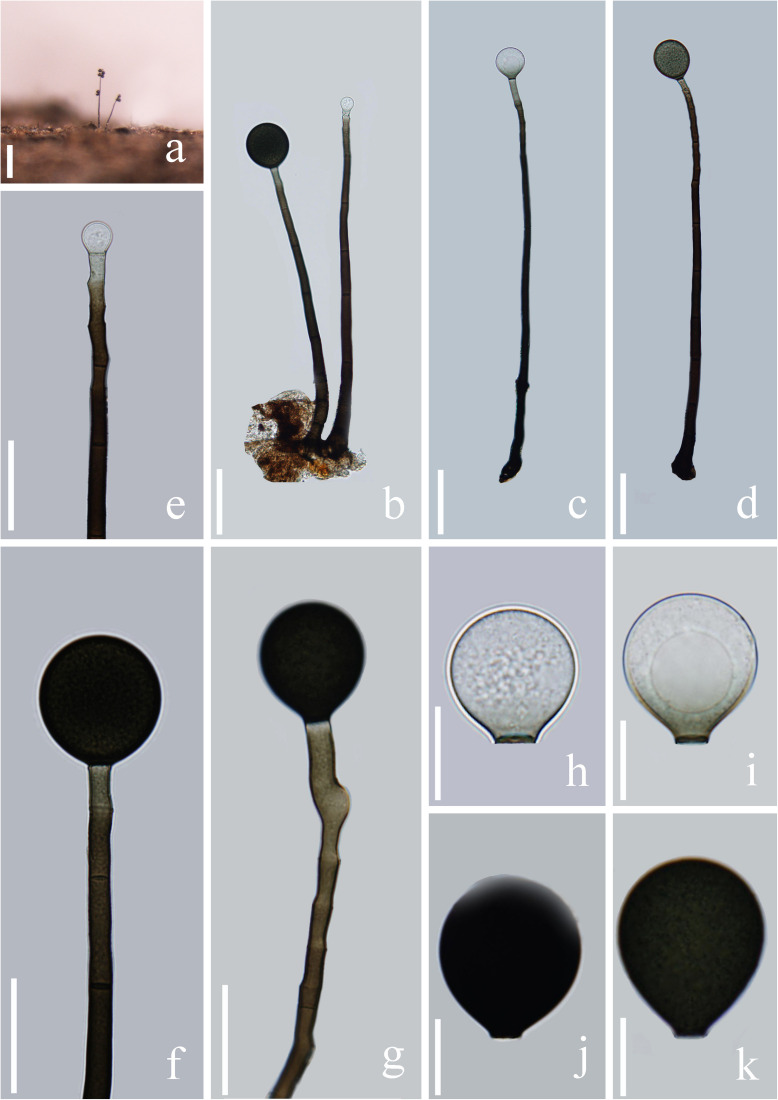
*Acrogenospora yunnanensis* (MFLU 20–0292, holotype) **(a)** Colony on wood. **(b–d)** Conidiophores, conidiogenous cells and conidia. **(e–g)** Conidiogenous cells and conidia. **(h–k)** Conidia. Scale bars: **(a)** 200 μm, **(b–d)** 50 μm, **(e–g)** 30 μm, **(h–j)** 15 μm.

*Index Fungorum number*: IF 557600; *Facesoffungi number*: FoF 07985, Figure 10

Holotype—MFLU 20–0292

Etymology—Referring to Yunnan province, China, where the fungus was collected.

*Saprobic* on submerged decaying wood. **Sexual morph:** Undetermined. **Asexual morph:** *Colonies* effuse on natural substrate, hairy, dark brown. *Mycelium* partly immersed, partly superficial, composed of septate, brown to dark brown, branched, smooth hyphae. *Conidiophores* 260–390 × 8.5–12 μm ($\bar{x}$ = 326 × 10.3 μm, *n* = 15), mononematous, macronematous, solitary, erect, straight or slightly flexuous, cylindrical, unbranched, brown to dark brown, paler toward apex, septate, smooth. *Conidiogenous cells* holoblastic, monoblastic, integrated, initially terminal, later becoming intercalary, cylindrical, smooth, pale brown, proliferating percurrently. *Conidia* 23–32.5 × 22–30 μm ($\bar{x}$ = 28 × 26 μm, *n* = 30), acropleurogenous, solitary, spherical or subspherical, truncate at base, aseptate, hyaline when young, dark brown to black when mature, smooth.

*Material examined*: CHINA, Yunnan Province, Laojunshan Mountain, on decaying wood submerged in a stream, August 2015, A.L. Shi, S-1114 (MFLU 20–0292, **holotype**); ex-type culture, MFLUCC 18–1611. CHINA, Gaoligingshan mountain, saprobic on decaying wood submerged in a stream, August 2015, A.L. Shi. S-774 (DLU 774, **isotype**), living culture, MFLUCC 20–0099.

*Notes*: In the phylogenetic analysis, *Acrogenospora yunnanensis* shares a sister relationship to *A. submersa*. Morphologically, *A. yunnanensis* can be distinguished from *A. submersa* by the longer conidiophores (Table 2) and color of conidia. *Acrogenospora yunnanensis* has dark brown to black conidia with a large guttule, while conidia of *A. submersa* are pale orange-brown to olivaceous brown at maturity and lack a guttule.

Morphologically, *A. yunnanensis* is most similar to *A. gigantospora* and *A. subprolata* in having similar conidial shape. However, they differ in size of conidiophores and conidia (Table 2).

## Discussion

In this study, we provide new descriptions and illustrations for seven new species and two known species of *Acrogenospora*. This study contributes to a better taxonomic understanding and proposes that there could be a number of additional new species within the genus and its diversity could be much higher than anticipated. *Acrogenospora* species are cosmopolitan with worldwide distribution, they are mainly found on dead and submerged wood especially in freshwater habitats (Hughes, 1978; Goh et al., 1998; Ma et al., 2012; Hyde et al., 2019). Among the 20 *Acrogenospora* spp., 15 species were reported from freshwater habitats and only 5 of them were recovered from terrestrial habitats. Our study has shown that in a small area of Yunnan Province there are 14 species of *Acrogenospora* in streams alone and indicates that the genus is highly diverse, and has been found to occur with other genera in the region (Hyde et al., 2019). Previous studies have also reported that there could be an amazing fungal diversity hidden in the South East Asian region (Hyde et al., 2018).

*Acrogenospora* species are quite similar to each other, and previous studies suggested to distinguish them based on conidial shape, size, and color and the degree of pigmentation of the conidiophores (Hughes, 1978, Table 2). We found that guttules and basal cells of conidia are also important characters to distinguish species and a morphological comparison of all *Acrogenospora* species is provided (Table 2).

Previous publications on submerged wood in freshwater have lumped several *Acrogenospora* collections and identified them based on morphology as *A. sphaerocephala* (Table 3) perhaps because of the difficulty of using morphs alone to delineate species and due to a lack of DNA sequence data. It is likely that the collections of *A. sphaerocephala* in older publications (Table 3) are wrongly named and further taxonomic work is necessary.

**TABLE 3:** Collections of *Acrogenospora* from freshwater habitats.

*Acrogenospora* collections	Location	Habitat	Host	References
*A. altissima*	New Zealand	Lake	Rotten wood of *Weinmannia racemosa*	Goh et al., 1998
*A. aquatica*	China (Yunnan)	Stream	Submerged wood	This study
*A. basalicellularispora*	China (Yunnan)	Stream	Submerged wood	This study
*A. ellipsoidea*	China (Yunnan)	Stream	Submerged wood	Hu et al., 2010
*A. guttulatispora*	China (Yunnan)	Stream	Submerged wood	This study
*A. gigantospora*	New Zealand	Lake	Rotten wood of *Weinmannia racemosa*	Hughes, 1978
*A. obovoidspora*	China (Yunnan)	Stream	Submerged wood	This study
*A. olivaceospora*	China (Yunnan)	Stream	Submerged wood	This study
*A. ovalia*	China (Hongkong)	Reservoir	Submerged wood	Goh et al., 1998
*A. sphaerocephala*	China (Hongkong)	Stream	Submerged wood	Tsui et al., 2000
*A. sphaerocephala*	Philippines	Stream	Submerged wood and bamboo	Cai et al., 2003
*A. sphaerocephala*	China (Hongkong)	Stream	Submerged wood and *Pinus* baits	Ho et al., 2001
*A. sphaerocephala*	Durban, South Africa	River	Submerged *Phragmites*	Hyde et al., 1998
*A. sphaerocephala*	Seychelles	River	Submerged wood	Goh et al., 1998
*A. sphaerocephala*	China (Hongkong)	Reservoir	Submerged wood	Goh and Hyde, 1999
*A. sphaerocephala*	Australia	River	Submerged wood	Goh et al., 1998
*A. sphaerocephala*	UK	River	Submerged wood	Goh et al., 1998
*A. submersa*	China (Yunnan)	River	Submerged wood	This study
*A. subprolata*	China (Tibet)	Stream	Submerged wood	This study
*A. subprolata*	China (Hongkong)	River	Submerged wood	Goh et al., 1998
*A. thailandica*	Thailand	Stream	Submerged wood	Hyde et al., 2019
*A. verrucispora*	China (Yunnan)	Stream	Submerged bamboo culms	Zhu et al., 2005
*A. verrucispora*	China (Yunnan)	Stream	Submerged wood	This study
*A. yunnanensis*	China (Yunnan)	Stream	Submerged wood	This study

Before this study, there were 13 species of *Acrogenospora* but only three of them had sequence data available in GenBank, and there was no data for the ex-type strains. Our phylogenetic sampling included 12 strains of *Acrogenospora* (Acrogenosporaceae), and all strains grouped with four species of *Minutisphaera* (Minutisphaeraceae) within Minutisphaerales (99% ML and 0.99 PP, Figure 1). The results were similar to the analyses by Jayasiri et al. (2018). In our analyses, *Acrogenospora carmichaeliana* (CBS 206.36) did not cluster with other strains of *A. carmichaeliana*, instead clustered with our new isolate *A. basalicellularispora* with low statistical support. Unfortunately, there are no morphological descriptions for CBS 206.36, so we are unable to compare its morphology with our new isolate. Further collections and phylogenetic studies of *Acrogenospora* are needed to better understand the phylogenetic placement of those species which lack sequence data and, undoubtedly, many more novel species can be found.

The phylogenetic analysis provide clear resolution to the taxonomic complexities within this group. Protein-coding genes have been shown to be essential to identify a taxon up to species level (Tang et al., 2007, 2009; Jeewon et al., 2017). In our study, we sequenced the RPB2 and TEF1α sequence data to distinguish *Acrogenospora* species and the phylogenetic trees are provided in Supplementary Files 1, 2. In our phylogenetic tree (LSU + SSU + TEF1α + RPB2, Figure 1), *Acrogenospora aquatica*, *A. guttulatispora*, *A. submersa* and *A. yunnanensis* grouped together, but they constitute different clades based on phylogenies derived from the TEF and RPB2 data which clearly support that they are phylogenetically distinct species. *Acrogenospora verrucispora* clustered with *A. carmichaeliana* (Figure 1), but there are 9 bp differences in TEF1α gene region. In addition, they can be easily distinguished from each other by the shape, color and wall of conidia, (conidia of *A. verrucispora* are spherical or subspherical, orange-brown to olivaceous brown, distinctly verrucose-walled, while *A. carmichaeliana* has broadly ellipsoidal to obovoid, brown to dark brown, smooth-walled conidia). *Acrogenospora olivaceospora* is close to *A. sphaerocephala*, however, there are 12.3% nucleotide differences in RPB2 gene region between them. These results support our establishment of the new taxon as recommended by Jeewon and Hyde (2016). As for *A. basalicellularispora* and *A. subprolata* there are no DNA sequences available from protein-coding gene but they can be easily distinguished from other species based on morphological characters (Table 2).

Hyde et al. (2019) discussed whether *Acrogenospora sphaerocephala* was the asexual morph of *Farlowiella carmichaeliana* and whether *A. megalospora* was wrongly introduced as the asexual morph of *F. carmichaeliana*. In our phylogenetic analyses, *Acrogenospora sphaerocephala* did not cluster with *A. carmichaeliana*, forming different clades within Acrogenosporaceae. DNA sequences of Ex-type strains of both *A. megalospora* and *Farlowiella carmichaeliana* are unavailable in GenBank. Therefore, the connection of sexual and asexual morph of *Farlowiella carmichaeliana* is not clear and this needs further morpho-molecular evidence.

## Data Availability Statement

The datasets presented in this study can be found in online repositories. The names of the repository/repositories and accession number(s) can be found in the article/Supplementary Material.

## Author Contributions

D-FB conducted the experiments, analyzed the data, and wrote the manuscript. EM, DB, and KH revised the manuscript. Z-LL planned the experiments and analyzed the data. H-YS planned the experiments and funded the experiments. H-WS conducted the experiments. All authors revised the manuscript.

## Conflict of Interest

EM was employed by Manaaki Whenua Landcare Research. The remaining authors declare that the research was conducted in the absence of any commercial or financial relationships that could be construed as a potential conflict of interest.

## Footnotes

1 http://mafft.cbrc.jp/alignment/server/index.html

2 http://www.phylo.org/portal2/

3 https://www.treebase.org/

## Supplementary Material

FILE S1 Phylogenetic tree based on RAxML analysis of TEF1α sequence data. Bootstrap support values for maximum likelihood and maximum parsimony (MP, red) higher than 75% are indicated above the nodes as MLBS/MPBS. The tree is rooted with *Hysterographium fraxini* (MFLU 15-3035 and MFLU 15-3681).

FILE S2 Phylogenetic tree based on RAxML analysis of RPB2 sequence data. Bootstrap support values for maximum likelihood and maximum parsimony (MP, red) higher than 75% are indicated above the nodes as MLBS/MPBS. The tree is rooted with *Aliquandostipite khaoyaiensis* (AFTOL-ID 1364).
