# Supplementary figures and images for: Acrogenospora (Acrogenosporaceae, Minutisphaerales) Appears to Be a Very Diverse Genus
Source: Front Microbiol. 2020 Jul 24;11:1606. doi: 10.3389/fmicb.2020.01606 (PMC7393737; doi:10.3389/fmicb.2020.01606)

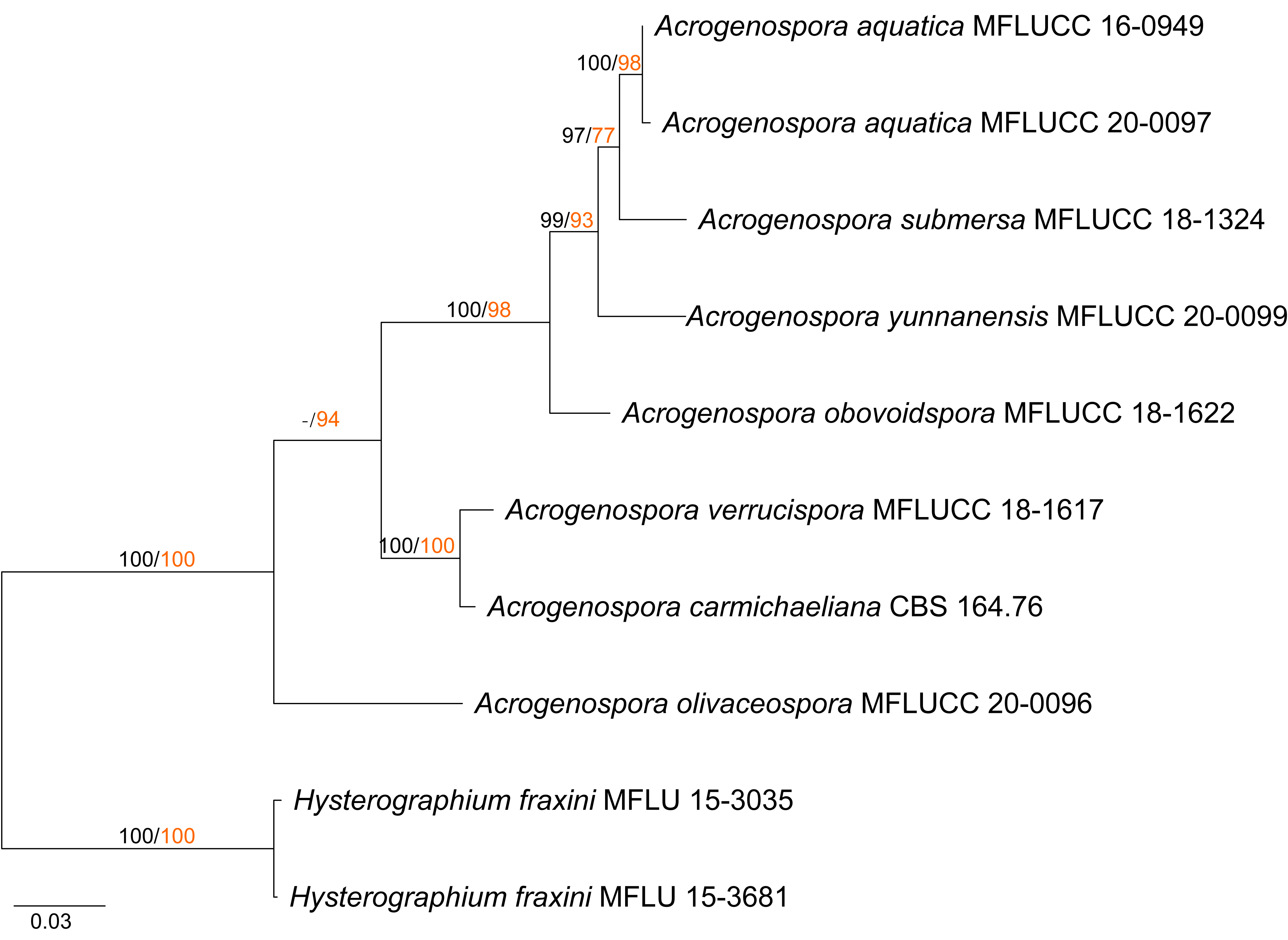

Supplement: FILE S1 — Phylogenetic tree based on RAxML analysis of TEF1α sequence data. Bootstrap support values for maximum likelihood and maximum parsimony (MP, red) higher than 75% are indicated above the nodes as MLBS/MPBS. The tree is rooted with Hysterographium fraxini (MFLU 15-3035 and MFLU 15-3681). [file Image_1.jpeg]

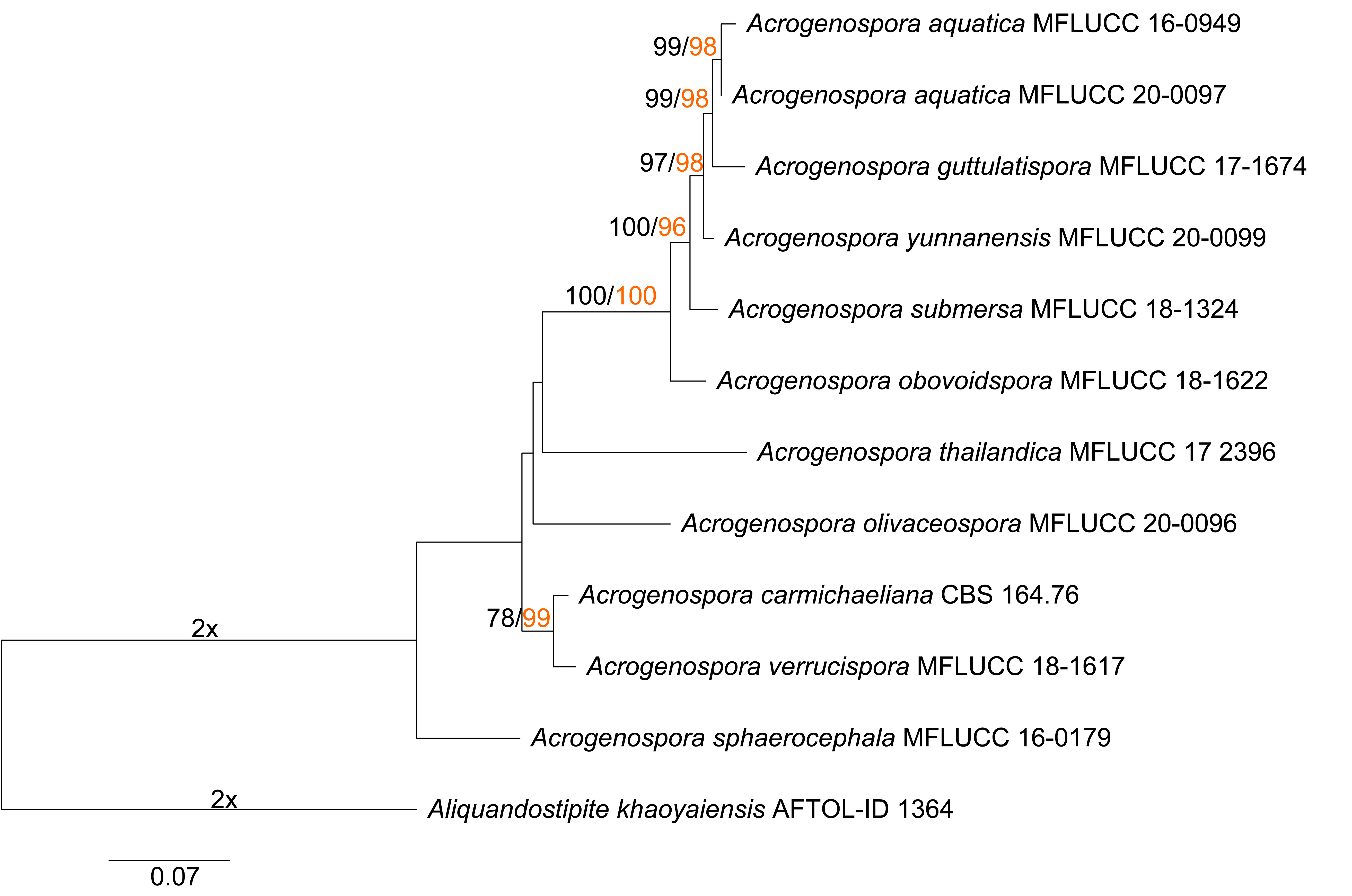

Supplement: FILE S2 — Phylogenetic tree based on RAxML analysis of RPB2 sequence data. Bootstrap support values for maximum likelihood and maximum parsimony (MP, red) higher than 75% are indicated above the nodes as MLBS/MPBS. The tree is rooted with Aliquandostipite khaoyaiensis (AFTOL-ID 1364). [file Image_2.jpeg]
